# NLRP3 Inflammasome Role and NLRP3 Inhibitors in Sensorineural Hearing Loss

**DOI:** 10.3390/biom16020186

**Published:** 2026-01-26

**Authors:** Mireia Toledano-Pinedo, José Marco-Contelles, Alexey V. Dobrydnev

**Affiliations:** 1 Laboratory of Medicinal Chemistry, Institute of General Organic Chemistry (CSIC), C/Juan de la Cierva 3, 28006 Madrid, Spain; mireia.toledano@iqog.csic.es; 2 Chemistry Department, Taras Shevchenko National University of Kyiv, Volodymyrska Street 60, 01033 Kyiv, Ukraine; alexey.pierrot@gmail.com

**Keywords:** anakinra, canakinumab, cryopyrin-associated periodic syndromes, deafness, hearing loss, inflammasome, MCC950, NLRP3, oridonin, piceatannol

## Abstract

Herein, we will review the function of the NLRP3 inflammasome in hearing loss (HL), summarize the most significant experimental results described in recent years, describe the biological mechanisms involved in the role of NLRP3 inflammasome in HL, and highlight some of the most promising small NLRP3 inhibitors for its clinical therapy, showing excellent pharmacological effects and good pharmacodynamic/pharmacokinetic profiles.

## 1. Introduction

The inflammasome is a multiprotein complex located in the cell cytoplasm that regulates the innate immune system [1]. Over the last decade, considerable interest has been focused on the NLR family pyrin domain-containing 3 (NLRP3) inflammasome as a key biological actor associated with a number of pathologies, influencing different physiological and pathological events [1], including neurodegenerative disorders such as Alzheimer’s disease [2]. Furthermore, the inflammasome also plays a crucial role in the pathological progression of hearing loss (HL) and noise-induced hearing loss (NIHL). In spite of this, the precise contribution and mechanistic involvement of NLRP3 inflammasome activation in HL and NIHL remain incompletely defined [1]. Currently, the therapy of NLRP3 inflammasome-mediated diseases consists of the use of anti-interleukin (IL-1β) antibodies, although drugs acting as specific NLRP3 inhibitors (NLRP3is) could be more appropriate than IL-1β antibodies.

In this study, we will review the role of the NLRP3 inflammasome and NLRP3 inhibitors in sensorineural hearing loss (SNHL) [2].

## 2. NLRP3 Inflammasome and Auto-Inflammatory Disorders

**Figure 1:**
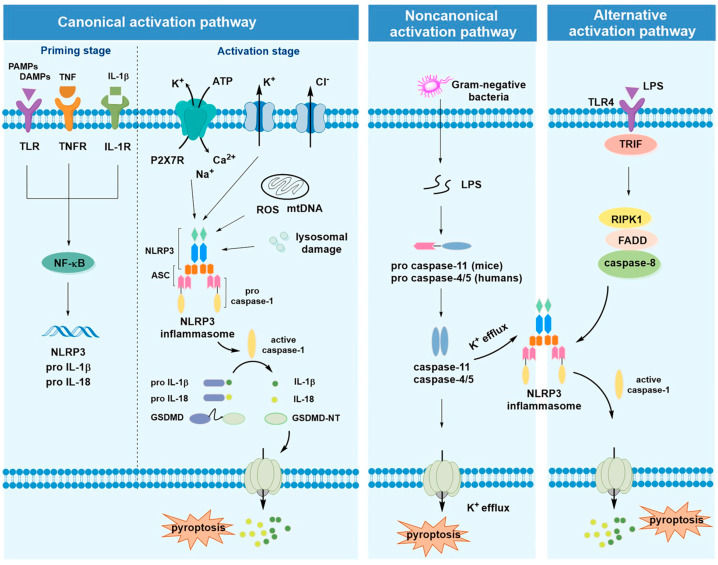
NLRP3 inflammasome activation mechanisms (canonical, noncanonical, and alternative pathways) (taken from reference [3] with permission).

The NLRP3 inflammasome is an intracellular innate immune sensor that is manifested in immune cells, such as monocytes or macrophages (Figure 1) [3]. Stimulation of the NLRP3 inflammasome produces IL-1β secretion, whereas mutations of NLRP3 give an unusual expression of the NLRP3 inflammasome, resulting in a spectrum of autosomal dominant systemic autoinflammatory disease (AID) known as cryopyrin-associated periodic syndrome (CAPS) [4].

Cryopyrin-associated periodic syndromes (CAPSs) comprise the familial cold auto-inflammatory syndrome (FCAS), Muckle–Wells syndrome (MWS), as well as the neonatal-onset multisystem inflammatory disease (NOMID), also known as chronic infantile neurological, cutaneous, and articular (CINCA) condition [5]. These syndromes share a similar mutation in the NLRP3 gene [5]. The modified gene product cryopyrin stimulates the inflammasome that is responsible for the abnormal formation of IL-1β, which leads to inflammatory events in CAPSs, such as enhanced SNHL, osteoarticular involvement, and central nervous system (CNS) evidence. Drug discovery of readily available IL-1 inhibitors (IL-1is) has provided safe and effective therapeutic alternatives for this condition. Needless to say, to prevent severe and life-threatening disease consequences, an appropriate assessment, as well as a rapid initiation of therapy, are critical for an effective outcome in most patients [5]. To sum up, CAPS is an inherited AID resulting from a gain-of-function mutation in NLRP3 [6,7], producing dysregulated liberation of IL-1β and hyperactivation of the inflammasome [8].

To test clinical, demographic, and genetic events in patients diagnosed with CAPS, all patients carrying NLRP3 variants have been independently validated, including those showing a damaging germline mutation. As a result, a large number of patients (136) were included in the study. The median age at clinical onset was nine months, with a median follow-up duration of 15 years. Musculoskeletal impairment, skin rash, and fever were the most commonly observed events. Neurological effects were noticed with ophthalmological implications in 71% of the patients, and neurosensory HL in 42% of the patients. Genetic analysis revealed heterozygous germline mutations in 133 patients, whereas three individuals were mutation-negative. A total of 31 different mutations of NLRP3 were registered; 7 rare variants were found in 78% of patients, and 24 accounted for 27 patients associated with early disease onset, neurological symptoms, and severe musculoskeletal implications. The T348M variant was linked to early disease onset, chronic development, and HL. Neurological implications were less strongly associated with the V198M, E311K, and A439V alleles. To sum up, patients showing rare NLRP3 variants are at risk of severe CAPS; beginning before the age of six months is linked to more severe neurological complications and HL [9].

Although CAPS patients currently endure SNHL, it remains unclear whether the CAPS-associated mutation in NLRP3 is affiliated with the development of HL [6]. However, patients diagnosed with CAPS show enhanced SNHL attributable to cochlear auto-inflammation, which, in rare cases, may represent the sole or predominant clinical manifestation [8].

Cold-prompted urticaria represents one of the most common clinical manifestations of CAPS. Nevertheless, a study identified 14 patients harboring pathogenic alterations affecting the p.Tyr861 residue within the LRR domain of NLRP3 who exhibited a low prevalence of cold-induced urticaria. This investigation aimed to explore a potential genotype–phenotype correlation in CAPS patients, along with analyzing at the cellular level the effect of the p.Tyr861Cys change on the activation of NLRP3 [7].

Clinical events in 14 CAPS patients carrying a heterozygous alteration at residue 861 within the LRR domain of NLRP3 were compared with those observed in 48 CAPS patients lacking this mutation. IL-1β production by peripheral blood mononuclear cells and isolated monocytes from affected individuals and healthy controls was evaluated following stimulation with lipopolysaccharide (LPS) and the crystals of monosodium urate. Patients harboring the residue 861 NLRP3 variant predominantly exhibited SNHL, while cutaneous urticarial manifestations were infrequent. Conversely, cells from patients with a pathogenic variant at position 861 required an activation signal to produce IL-1β. Still, they made more IL-1β during both the early and late phases of secretion compared with cells from well and fine donors. Pathogenetic modifications of Y861 in NLRP3 result in triggered hypersecretion of IL-1β associated with the atypical CAPS phenotype [7].

**Figure 2:**
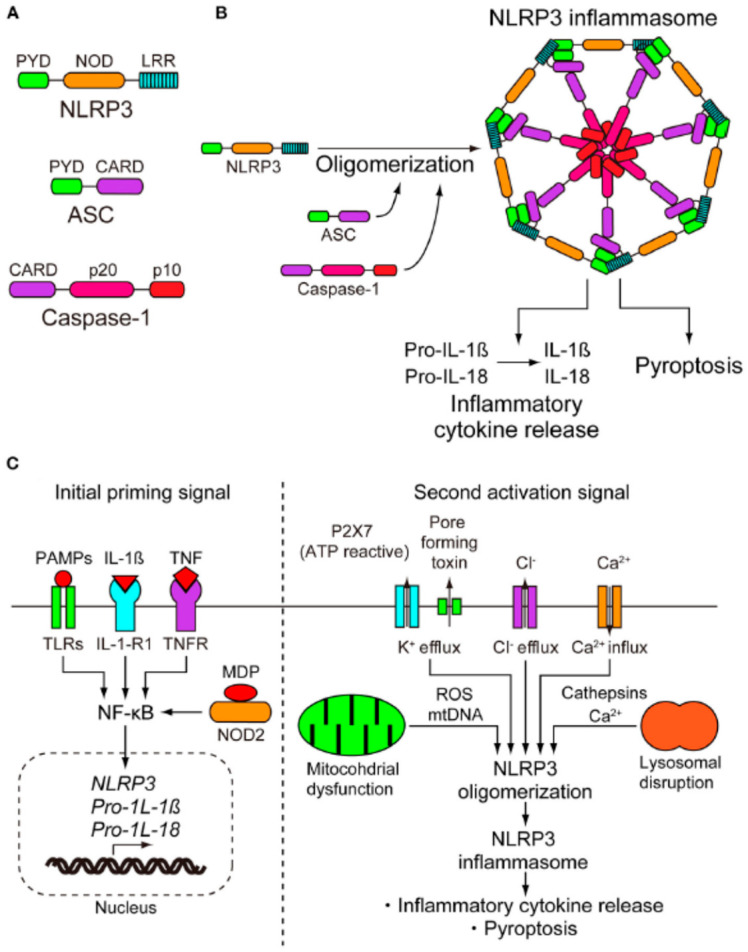
Activation of the NLRP3 inflammasome (taken with permission of the authors from ref. [10]).

Recent analyses have shown that modifications of the NLRP3 gene produce genetic disorders, such as CAPS and non-syndromic SNHL DFNA34. NLRP3 modifications facilitate CAPS and DFNA34 by stimulating the NLRP3 inflammasome and enhancing IL-1β production. Patients with CAPS and DFNA34 manifest progressive bilateral SNHL. HL has specific features that the anti-IL-1 therapy can treat. However, it is not easy to improve genetic HL with drugs [10]. It should be observed that there are potentially numerous outcomes in response to the therapies. Therefore, a thorough understanding of the clinical characteristics of CAPS and DFNA34, together with comprehensive NLRP3 mutation analysis, is essential for early recognition and definitive diagnosis (Figure 2) [10].

IL-1is, such as **anakinra** and **canakinumab,** have provided, for the first time, an efficient therapeutic option to treat CAPS [4]. **Canakinumab** is a totally human IgG1 monoclonal antibody targeting IL-1β that provides selective and sustained inhibition of IL-1β signaling, resulting in rapid and robust clinical responses in most patients with CAPS, with a favorable safety profile and minimal secondary complications. Long-term follow-up studies have demonstrated sustained efficacy, safety, and tolerability of the treatment [5]. Accordingly, **canakinumab** received approval from the U.S. Food and Drug Administration for the treatment of FCAS and MWS, and from the European Medicines Agency for the management of all three CAPS phenotypes [5].

**Muckle–Wells syndrome** (**MWS**). MWS is an AID showing rash, fever, arthralgia, amyloidosis, and sensorineural deafness. The E311K variant in NLRP3/CIAS1 resulted in phenotypic heterogeneity among individuals with MWS [11,12]. In 2012, the first analysis describing the pretreatment otological status of subjects with familial MWS was reported [13]. Thus, a single-center cohort was analyzed using audiological and neurotological tests, including pure tone audiograms and tinnitus assessments. Audiograms from members of the same families were compared to assess family-specific risk for the development of HL. A total of 19 patients (aged 3–72 years) from four families carrying three distinct NLRP3 gene mutations were included in the analysis. Most patients (89%; 17 of 19) showing bilateral SNHL initially presented with high-frequency HL, which progressed to significant deafness in the most severe cases. The vestibular caloric responses remained largely intact even in individuals with severe HL. Nearly 50% of participants experienced intermittent or chronic tinnitus [13,14].

A study was conducted to evaluate the clinical features and therapeutic effects of treatment with IL-1 inhibitors, such as **anakinra** or **canakinumab** [11]. Initially, 42 patients and family members were screened for the presence of NLRP3 mutations, and clinical features were systematically reviewed in all participants. Laboratory assessments included measurements of erythrocyte sedimentation rate, C-reactive protein, and serum amyloid A (SAA), as well as cytokine and cytokine receptor levels. Thirteen patients were identified as heterozygous carriers of the p.Glu311Lys (E311K) amino acid substitution encoded by exon 3 of the NLRP3 gene, whereas none of the unaffected family members carried this variant. Clinical manifestations exhibited marked heterogeneity; however, with the exception of one pediatric case, all mutation carriers experienced hearing loss and pronounced fatigue. Elevated levels of TNF-α, IL-6, TNF-RI, TNF-RII, and SAA were detected in three, two, one, six, and ten patients, respectively. Both clinical symptoms and laboratory abnormalities showed rapid improvement following treatment with anakinra or **canakinumab**. To summarize, the NLRP3 E311K mutation is associated with a heterogeneous clinical spectrum, which may enhance our understanding of MWS presentation. The most critical clinical evidence was HL. Pericarditis, a rare but significant clinical sign of MWS, was diagnosed in three patients. One patient had a severe course, which led to renal failure secondary to amyloidosis [11].

**Meniere’s disease** (**MD**). MD is a multifactorial pathology of the inner ear resulting in HL. Although the function of immune reactions in MD has been suggested, their accurate mechanisms are uncertain. A recent study demonstrated that the downregulation of serum/glucocorticoid-inducible kinase 1 (SGK1) is associated with enhanced activation of the NLRP3 inflammasome in vestibular resident macrophage-like cells derived from MD patients [15]. Depletion of SGK1 markedly increased interleukin-1β (IL-1β) production, resulting in injury to inner ear hair cells and the vestibular nerve. Mechanistically, SGK1 was shown to interact with the pyrin domain (PYD) of NLRP3 and to phosphorylate it at serine 5, thereby promoting inflammasome assembly. Sgk-/- mice exhibited pronounced audiovestibular abnormalities and heightened inflammasome activation in a lipopolysaccharide-induced endolymphatic hydrops model, effects that were attenuated by NLRP3 inhibition. Consistently, pharmacological inhibition of SGK1 exacerbated disease severity in vivo. To summarize, these results confirm that serum/glucocorticoid-inducible kinase 1 stimulates NLRP3 and preserves inner ear immune homeostasis, thereby participating in models of MD pathology [15].

## 3. Hearing Loss and NLRP3

Age-related hearing loss (ARHL) or presbycusis is a progressive loss of hearing sensitivity primarily associated with the degeneration of sensory or transduction neurons in the peripheral and central auditory systems. Increased generation of reactive oxygen species (ROS) is commonly observed in aging cochleae. Moreover, inflammasome activity has been implicated as a potential contributor to elevated ROS production in immune cells. Nevertheless, it remains unclear whether inflammasomes play a direct role in the pathogenesis and progression of ARHL [16].

In a recent study, miniature pigs were exposed to white noise at an intensity of 120 dB, and auditory brainstem response (ABR) measurements were used to assess auditory function. Immunofluorescence staining, Western blot (WB) analysis, confocal laser scanning microscopy, and quantitative reverse transcription–polymerase chain reaction (qRT-PCR) were employed to assess the localization and expression of inflammasome-related proteins. Following noise exposure, the cochleae exhibited markedly increased expression of NLRP3, IL-18, IL-1β, and cleaved caspase-1. These findings suggest that the activation of the NLRP3 inflammasome within the cochlea occurs in response to acoustic trauma and may represent a key pathogenic mechanism underlying noise-induced hearing loss (HL) [1].

When activated by unconjugated bilirubin (UCB), inflammatory intermediaries, namely IL-18 and TNF, promote the neurotoxicity and ototoxicity found in severe neonatal hyperbilirubinemia [17]. However, at the cell and molecular level, the ruling and mechanism of UCB-prompted ototoxicity is unknown. In a very recent report, seven-day-old mammary rats were exposed to different doses of UCB to reproduce infant auditory injury. The ABR result showed significant HL, which took place with enhanced concentrations. Morphological analysis of organotypic cochlear cultures treated with diverse concentrations of UCB showed that auditory nerve fibers were demyelinated, and the diameters of spiral ganglion neurons (SGN) were reduced. Furthermore, house ear institute-organ of Corti 1 (HEI-OC-1) cells treated with various doses of UCB indicated necrosis by flow cytometry. The morphological feature of pyroptosis has been observed using a scanning electron microscope. Cleaved caspase-1, gasdermin D (GSDMD), and NLRP3 expression were significantly enhanced in cochlear explants with UCB-induced pyroptosis. Furthermore, to elucidate the molecular mechanisms underlying UCB-induced pyroptosis in inner ear cells, specific inhibitors of pyroptotic pathways were employed. Treatment with these inhibitors resulted in significantly reduced levels of pyroptosis-associated proteins, including cleaved caspase-1, GSDMD, ASC, IL-18, and NLRP3, compared with cells exposed to UCB alone. To summarize, these results suggest that the ERK/NLRP3/GSDMD signaling pathway is involved in UCB-induced ototoxicity [17].

Recent reports have identified a missense mutation in the NLRP3 gene associated with autosomal dominant SNHL accompanied by cochlear autoinflammatory pathology in two unrelated families. The NLRP3 gene encodes the NLRP3 protein, a core part of the NLRP3 inflammasome, which is predominantly expressed in innate immune cells, including macrophages and monocytes. Gain-of-function mutations in NLRP3 result in aberrant activation of the inflammasome and excessive release of IL-1β, giving rise to a range of autosomal dominant systemic autoinflammatory phenotypes [18]. Notably, affected individuals in these families displayed atypical clinical phenotypes that differed from those classically observed in CAPS. Experimental evidence further demonstrates that the inflammasome can be activated in macrophage/monocyte-like cells within the murine cochlea, leading to IL-1β production. Moreover, these cochlear macrophage/monocyte-like cells have been implicated in the development of hearing loss in a Slc26a4-deficient mouse model of human deafness [18].

Cytomegalovirus (CMV)-associated SNHL represents a significant global health burden. Recent studies have reported a correlation between the extent of SGN loss and the severity of HL following CMV infection. However, the molecular mechanisms underlying CMV-induced SGN degeneration remain poorly understood, underscoring the urgent need for effective therapeutic strategies [19,20]. In a recent report, it has been described that CMV-induced SGN death involves both apoptosis and pyroptosis, as a consequence of the simultaneous stimulation of the p53/JNK and NLRP3/caspase-1 signaling routes [19]. Furthermore, given that members of the mixed lineage kinase (MLK1/2/3) family are involved in host antiviral defense and function as upstream regulators of both p53/JNK and inflammatory signaling pathways, it has been confirmed that the pharmacological inhibition of MLKs with **URMC-099** protected against CMV-induced SGN death and HL. These findings identify MLK signaling as a critical and promising therapeutic target for suppressing apoptosis and pyroptosis during CMV infection in SGN cells, as well as for the therapy of HL [19]. In the same area of research, Zhuang et al. described an experimental model of HL in murine CMV (MCMV) in neonatal mice [20]. ABR testing was performed at three weeks post-infection to assess auditory function. The expression of inflammasome-associated factors was evaluated by immunofluorescence, WB analysis, qRT-PCR, and ELISA. MCMV sequentially induced inflammasome-associated events, and the inflammasome-associated factors were also enhanced in cultured SGN infected with MCMV for 24 h. Furthermore, MCMV enhanced the ROS. Collectively, these findings suggest that MCMV-induced HL is associated with ROS-driven inflammatory responses [20].

Cisplatin-induced ototoxicity is a severe adverse effect commonly observed following antitumor chemotherapy [21]. To investigate the role and underlying mechanisms of Pou4f3 gene mutations in cochlear pyroptosis, mice were administered cisplatin intraperitoneally to establish an experimental model of hearing loss, and Pou4f3 expression was manipulated using sh-Pou4f3 and mutant expression vectors. Apoptosis of cochlear hair cells was assessed by TUNEL staining, inflammatory responses were quantified using ELISA, and the expression of pyroptosis-related proteins was evaluated by immunofluorescence and WB analysis. Cisplatin exposure induced cochlear hair cell pyroptosis through the activation of the NLRP3/caspase-3/gasdermin E (GSDME) signaling pathway and was associated with a marked downregulation of Pou4f3 expression. Moreover, Pou4f3 mutations promoted cochlear hair cell pyroptosis by activating the same NLRP3/caspase-3/GSDME pathway. Knockdown of Pou4f3 can enhance the effect of cisplatin treatment to induce pyroptosis of cochlear hair cells through the NLRP3/caspase-3/GSDME pathway. Consequently, it was concluded that the Pou4f3 gene mutation induces the pyroptosis of cochleae in cisplatin-prompted deafness mice through the NLRP3/caspase-3/GSDME pathway [21].

In an investigation aimed at elucidating the mechanisms underlying cisplatin-induced ototoxicity, a study was conducted to examine the inflammatory processes affecting the cochlear *stria vascularis* and the associated injury to marginal cells (MCs), which remain incompletely understood [22]. An in vitro model of cisplatin-induced MC damage was established, and analyses by PCR and WB revealed significantly increased expression of NLRP3, GSDMD, IL-1β, and caspase-1 in MCs. Targeted downregulation of NLRP3 using interfering RNA markedly attenuated cisplatin-induced MC pyroptosis. Moreover, the suppression of thioredoxin-interacting protein (TXNIP) expression inhibited NLRP3 inflammasome activation and the associated pyroptotic response. To summarize, these findings demonstrate that activation of the NLRP3 inflammasome may cause cisplatin-induced MC pyroptosis in cochlear *stria vascularis* and identify TXNIP as a possible upstream regulator and a potential attractive agent for treating cisplatin-mediated HL [22].

Noise pollution represents a serious public health challenge. It was shown that chronic environmental noise exposure can induce reorganization of the auditory cortex and lead to behavioral abnormalities. Nevertheless, the impact of prolonged environmental noise exposure on inner ear pathology and the progression of HL remains poorly defined [23]. In a recent study, Feng and colleagues simulated ecological noise exposure using prolonged white noise at a sound pressure level of 70 dB and examined its impact on the inner ears of C57BL/6J mice, while also establishing an in vitro model to investigate the underlying mechanisms. Their findings demonstrated that environmental noise exposure elevated auditory thresholds, reduced auditory response amplitudes, and aggravated both the severity and extent of ARHL, particularly within the intermediate frequency range. The cochlear ribbon synapse has been identified as a primary site of inner ear damage induced by environmental noise exposure [23]. In vitro simulations of glutamate-induced excitatory toxicity and aging-related stress demonstrated that activation of the NLRP3 inflammasome plays a critical role in cochlear ribbon synaptic injury. These findings indicate that prolonged exposure to low-intensity environmental noise can lead to HL through the disruption of ribbon synapses, likely mediated by inflammatory processes. Moreover, ecological noise exposure was shown to exacerbate the progression of ARHL. Collectively, this work elucidates key pathogenic mechanisms underlying environmental noise-induced inner ear injury and suggests potential avenues for the prevention and treatment of HL [23].

Vestibular schwannoma (VS) is one of the most common intracranial tumors, which arises from neoplastic Schwann cells of the vestibular nerve, producing debilitating SNHL and tinnitus [24]. Although VS-associated SNHL is attributed not only to mechanical compression of the auditory nerve but also to intrinsic biological characteristics of the tumor, it has been hypothesized that patients with VS-related hearing impairment exhibit a more pronounced inflammatory response than those with preserved hearing [24]. In this context, Sagers and colleagues analyzed microarray data from 80 VS samples and 16 healthy peripheral nerve specimens, identifying the NLRP3 inflammasome as a novel target warranting further investigation. These findings were subsequently validated at both the transcriptional and protein levels in human VS tissues using qRT-PCR and immunohistochemistry. Although activation of the NLRP3 inflammasome has not previously been described in VS, this finding suggests new therapeutic opportunities. Notably, analysis of 30 VS specimens revealed that the overexpression of key NLRP3 inflammasome components was significantly associated with tumors causing more severe HL in affected patients. Collectively, the therapeutic strategies for VS aimed at attenuating NLRP3-mediated inflammation may be beneficial for preserving auditory function [24].

Nonsyndromic HL is genetically heterogeneous yet phenotypically similar across many cases. Although several targeted next-generation sequencing (NGS) panels have been developed to facilitate the genetic diagnosis of nonsyndromic deafness, pathogenic variants in syndromic deafness-associated genes that are not included in these panels may give rise to clinical phenotypes resembling nonsyndromic HL [25]. In an effort to clarify this issue, Chen et al. conducted a comprehensive genetic evaluation of an autosomal dominant family where the proband had originally been diagnosed with nonsyndromic deafness [25]. Targeted NGS of 72 nonsyndromic and 72 syndromic deafness-related genes failed to identify pathogenic variants. Subsequent whole-exome sequencing revealed a p.Glu313Lys (E313K) mutation in NLRP3, a gene previously associated with MWS-related syndromic deafness but not included in earlier targeted deafness panels. Follow-up clinical evaluations identified only mild inflammatory manifestations in addition to HL in six of the nine affected individuals, whereas the remaining three showed no overt signs of MWS-associated inflammation. Furthermore, immunostaining of the murine cochlea demonstrated prominent NLRP3 expression in SGNs. Collectively, these findings suggest a specific role for NLRP3 in SGN function and indicate that NLRP3 variants may be implicated in both syndromic and nonsyndromic forms of sensorineural HL [25].

Sensorineural deafness is a prevalent global health issue; however, current therapeutic options are scarce [26]. Emerging evidence suggests that mitochondrial dysfunction plays a crucial role in the origin and development of deafness. ROS-induced mitochondrial dysfunction, combined with the stimulation of the NLRP3 inflammasome, is included in cochlear injury. Autophagy not only facilitates the removal of damaged proteins and dysfunctional mitochondria through mitophagy but also contributes to the reduction in intracellular ROS levels. Conversely, the upregulation of autophagic activity attenuates oxidative stress, suppresses apoptotic signaling, and protects auditory cells. These observations underscore the interconnected roles of ROS generation, NLRP3 inflammasome activation, and autophagy in the pathogenesis of HL, including noise-induced and ARHL [26].

The NLRP3 gene mutations are responsible for autosomal dominant autoinflammatory disorders collectively referred to as NLRP3-associated autoinflammatory disease (NLRP3-AID). Recent evidence indicates that HL may represent the sole or primary clinical manifestation of NLRP3-AID [27]. In a large-scale genetic analysis of 110 autosomal dominant HL families using a customized panel comprising 237 HL-associated genes, one family was identified as carrying the NLRP3 c.1872C>G (p.Ser624Arg) variant [27]. Functional characterization demonstrated that this novel mutation confers a gain-of-function effect, leading to increased caspase-1 activity and excessive production of the proinflammatory cytokine IL-1β. Clinical evaluation of affected individuals, supported by serological evidence of inflammation and pathological cochlear signal enhancement on FLAIR-MRI, was consistent with a diagnosis of NLRP3-AID. These findings highlight the importance of genetic testing in patients with progressive postlingual HL for identifying NLRP3-AID-associated hereditary HL and for enabling timely, targeted therapy with IL-1Ra [27].

AID is a rare disorder characterized by inflammation in the absence of antigen-specific T cells, resulting in neurological manifestations and HL [28]. Salsano and co-workers have reported on a 30-year-old man who had a chronic disease characterized by progressive HL, showing high inflammatory markers [28]. The hypothesis of an AID led to evaluating the potential mutations in IL-1 receptor-related genes, as well as functional analyses of IL-1 receptor activity in patient-derived mononuclear cells. Genetic screening of IL-1 pathway-associated genes identified a novel NLRP3 (CIAS1) variant (p.I288M) and a previously reported MEFV mutation (p.R761H); however, their combined presence was considered non-pathogenic. In addition, selective hypersecretion of IL-6 within the CNS was proposed as the primary pathogenic mechanism. It was supported by a favorable clinical response to the anti–IL-6 receptor monoclonal antibody **tocilizumab**, in contrast to the lack of response to the recombinant IL-1Ra **anakinra**. Whole-exome sequencing did not reveal pathogenic variants in other genes associated with AID. Consequently, it was proposed that the clinical presentation in this patient was considered representative of a novel category of AID by marked neurological manifestations [28].

The pathogenesis of HL in AID mediated by inflammasome activation remains incompletely understood. To address this gap, Kim and colleagues developed a novel mouse model harboring NLRP3 alterations that exhibited quantifiable HL in addition to other syndromic features and systematically analyzed audiological and histopathological changes in the cochlea to elucidate the mechanisms by which NLRP3 mutations lead to autoinflammatory HL [29]. To achieve cochlea-specific expression of mutant NLRP3, NLRP3 mutant mice were crossed with Gfi1 knock-in mice, enabling conditional activation of mutant NLRP3 in the inner ear and hematopoietic cells. Hearing thresholds were assessed, and hematoxylin and eosin-stained sections of the cochlea, brain, liver, and kidney were examined by microscopy. In addition, immunohistochemical analyses using polyclonal anti-NLRP3 antibodies were performed on cochlear whole-mount preparations and frozen sections. As a result, this study proposed, for the first time, a mouse model exhibiting quantifiable HL attributable to NLRP3 alterations. ABR recordings in NLRP3–Gfi1 mice, despite their limited lifespan, revealed moderate to severe HL by postnatal day 20. There was overall overexpression of mutant NLRP3 and mutant NLRP3 expression in the outer sulcus region, the spiral prominence, the inner sulcus, the organ of Corti, and the SGN in the cochlea. The hematoxylin–eosin-stained cochlear sections from NLRP3–Gfi1 mice at postnatal day 12 (P12) revealed disorganization of the organ of Corti between the outer hair cells and supporting Deiters’ cells, as well as the basilar membrane, compared with normal phenotype mice, resulting in the collapse of Nuel’s space. Notably, this morphological abnormality gradually returned to normal by P15. In addition, varying degrees of inflammation accompanied by lymphocytic infiltration were observed in the brain, kidney, and liver. To summarize, this study reports the first mouse model NLRP3–Gfi1 characterized by mutant NLRP3 overexpression and which demonstrated cochlear NLRP3 upregulation, a transient delay in cochlear development, and pronounced HL [29]. This mouse model, which recapitulates key features of human autoinflammatory HL, is anticipated to serve as a valuable tool for elucidating the pathogenic mechanisms underlying inflammasome activation-mediated auditory dysfunction [29].

## 4. NLRP3 Inhibitors as Drugs for Hearing Loss

AID produces HL, and it is known that inflammation produces HL in other pathologies. Resident macrophage populations within the inner ear respond to injurious stimuli, and their activation can lead to tissue damage [30]. The NLRP3 inflammasome is a multiprotein proinflammatory complex that has been activated in macrophages and implicated in the pathogenesis of HL. The aim of this section is to show that NLRP3 is a promising therapeutic target for SNHL across a spectrum of etiologies, varying from AID to tumor-associated HL in VS [30].

### 4.1. Piceatannol

**Figure 3:**
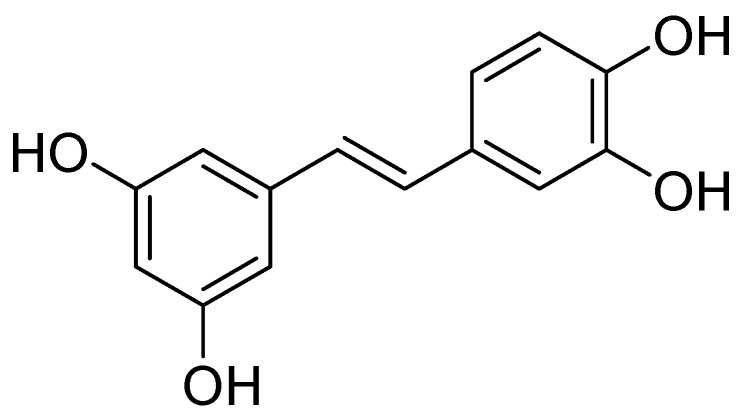
Structure of **piceatannol**.

A recent study has been dedicated to elucidating the mechanism underlying the protective action of **piceatannol** (**PCT**) (Figure 3) against inner ear hair cell damage associated with ARHL [31]. In vivo experiments confirmed that **PCT** confers protection against inflammation-driven, aging-related HL and SGN degeneration in mice. Moreover, treatment with the inflammatory vesicle inhibitor BAY11-7082 ameliorated ARHL, suppressed NLRP3 activation, and reduced gasdermin D (GSDMD) expression. In vitro experiments were conducted to simulate the aging inflammatory environment by applying LPS. The results indicated a marked increase in intracellular ROS levels, accompanied by the upregulation of caspase-11, NLRP3, and GSDMD expression. In contrast, treatment with PCT or BAY11-7082 significantly attenuated HEI-OC-1 cell injury while reducing inflammation-associated protein expression and pyroptotic activity. To summarize, these observations support ligand **PCT** as a protective agent against ARHL, acting through the caspase-11-GSDMD route, and thus represent a novel therapeutic option for HL.

### 4.2. Oridonin

**Figure 4:**
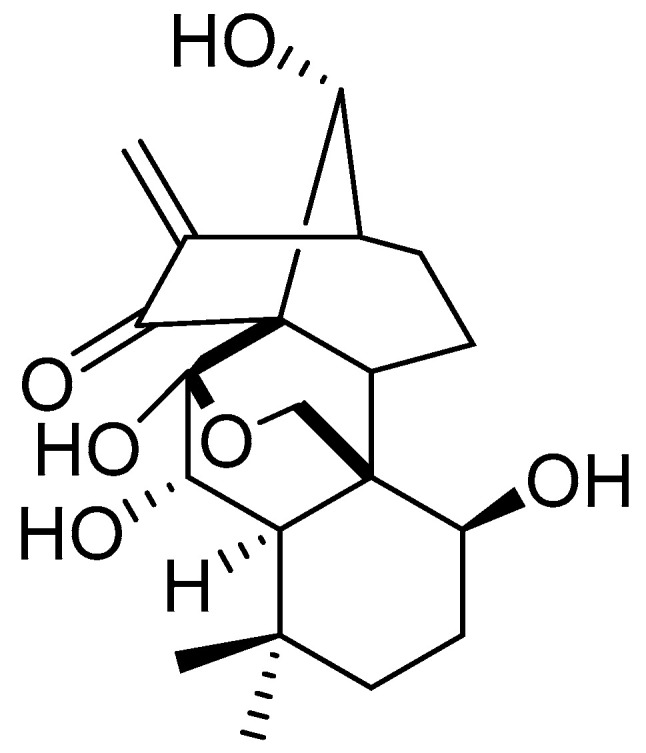
Structure of **oridonin**.

**Oridonin** (**Ori**) (Figure 4) has been described as a protective, anti-inflammatory hearing agent in mice following noise exposure by inhibiting the assembly of the NLRP3 inflammasome complex through disruption of the NLRP3–never in mitosis gene A-related kinase 7 (NEK7) interaction [32]. RNA sequencing analyses further indicated that interleukin-1 receptor type 2 (IL1R2) may represent a key downstream mediator of **Ori**-induced protection against NIHL. Consistently, IL1R2 expression was detected in SGN, as well as in inner and outer hair cells, in cochleae from **Ori**-treated mice. Furthermore, it was established that ectopic overexpression of IL1R2 in the inner ears of healthy mice using an adeno-associated virus delivery system significantly decreased noise-induced ribbon synapse injuries and HL by blocking the *cytokine storm* in the inner ear [32]. A recent study demonstrated that never in mitosis A (NIMA)-related kinase 7 (NEK7), a critical component and regulator of the NLRP3 inflammasome, directly interacts with NLRP3, and that this interaction can be disrupted by **Ori** [33]. Furthermore, treatment with Ori was shown to effectively block the NLRP3–NEK7 interaction and suppress downstream inflammasome activation in the cochleae of mice following noise exposure [33].

**Figure 5:**
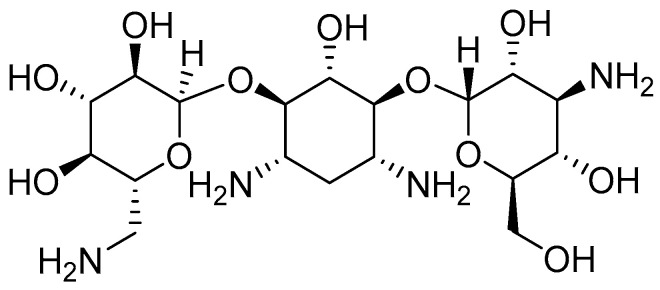
Structure of **kanamycin**.

Although the aminoglycoside antibiotic drugs produce HL, the pathological mechanisms are not yet known. In this context, accumulating evidence indicates that excessive ROS generation and inflammatory responses within the inner ear contribute to kanamycin (KM)-induced hair cell death and subsequent hearing loss (HL) (Figure 5). Accordingly, a recent study investigated the involvement of the NLRP3 inflammasome in KM-associated HL in mice [34]. The NLRP3 inhibitor **Ori** (Figure 4) was shown to markedly attenuate KM-induced HL by suppressing NLRP3 inflammasome activation and caspase-1/GSDMD-mediated hair cell pyroptosis. These findings suggest that both apoptotic and pyroptotic pathways contribute to KM-related HL and identify the NLRP3/caspase-1/GSDMD signaling axis as a promising therapeutic target for the treatment of aminoglycoside-induced hearing loss [34].

### 4.3. Anakinra

**Anakinra** (**Kineret**^®^) is a biopharmaceutical agent approved for the treatment of rheumatoid arthritis and CAPS and represents a slightly modified recombinant form of the human IL-1 receptor antagonist (IL-1Ra) protein [35]. A recent report has highlighted the potential therapeutic application of **anakinra**, an anti-IL-1 receptor antagonist, in selected cases of HL [36]. Though for severe HL that is not responsive to **anakinra**, cochlear implantation (CI) would be the last option for hearing rescue. The outcome of CI has not been clearly elucidated in this emerging type of HL associated with NLRP3-related AID, which has a systemic nature and a ubiquitous distribution of resident macrophages involving the SGN and the cochlear nerve. Thus, the successful outcome of CI in NLRP3-related AID has been reported [36]. Initially, three individuals representing distinct forms of NLRP3-related AID, CINCA, DFNA34, and MWS, subjected to CI for auditory rehabilitation, were enrolled. The clinical phenotypes and genotypes of these individuals were subsequently reviewed, and longitudinal audiological performance before and after CI was assessed. All three patients demonstrated favorable audiological outcomes, characterized by rapid improvement in speech perception test scores. Collectively, this study represents the first report describing CI outcomes in patients with confirmed NLRP3-related genetic etiologies and associated systemic inflammation, supporting CI as a viable therapeutic option in this population [36].

Gradually progressive SNHL is a common sensory disorder for which no curative treatment is currently available, rendering auditory rehabilitation with hearing aids or CI the primary management options. However, SNHL as a manifestation of the hereditary AID CAPS, or as the sole clinical feature of the cochlea-specific form (DFNA34), has been reported to respond to **anakinra** therapy, which improves the over-secretion of IL-1β induced by NLRP3 variants. Thus, the genotypic and phenotypic spectrum of CAPS or DFNA34 was investigated, with a specific focus on the responsiveness of SNHL to **anakinra** [35]. Seventeen families diagnosed with CAPS or DFNA34 were recruited for comprehensive evaluation. Genotypic and phenotypic assessments, including audiogram, MRI findings, and in vitro IL-1β assay, were performed. The cohort exhibited etiological homogeneity of 94.1% with respect to NLRP3 variants and a high rate of de novo occurrence (84.6%). A second DNFA34 pedigree worldwide was detected with a novel NLRP3 variant supported by in vitro testing. A substantial enhancement of hearing status, deviating from the natural course, was observed in three probands, including one with severe SNHL, in response to **anakinra**. A hearing threshold worse than 60 dB at the start of **anakinra** treatment and a cochlear increase on brain MRI appeared to be related to poor audiological prognosis and responsiveness to **anakinra** treatment, despite stabilized systemic symptoms and inflammatory markers. Consequently, several biomarkers, including NLRP3 genotypes, hearing status at diagnosis, and cochlear radiological findings, have been identified as prognostic factors for hearing status after **anakinra** therapy, and possibly as sensitive parameters for treatment dosage adjustment [35].

To determine whether the NLRP3 inflammasome contributes to the development of permanent NIHL, a recent study employed quantitative real-time polymerase chain reaction, WB, and ELISAs to assess inflammasome-related markers. The study demonstrated that expression levels of activated caspase-1, IL-18, IL-1β, and NLRP3 were noticeably enhanced in the cochleae of mice, which were exposed to broadband noise (120 dB) for 4 h as compared to the control animals [33]. These findings indicate robust inflammasome activation within the cochlea during NIHL pathogenesis and suggest that NLRP3, a sensor of ROS, plays a critical role in inflammasome assembly and subsequent inflammatory responses in the inner ear. In this context, treatment with **anakinra** was evaluated and found to partially attenuate hearing impairment at certain frequencies in an NIHL mouse model. Collectively, these results support the notion that the inhibition of NLRP3 inflammasome activation and its downstream signaling pathways may represent a promising therapeutic strategy for NIHL [33].

### 4.4. MCC950

**Figure 6:**
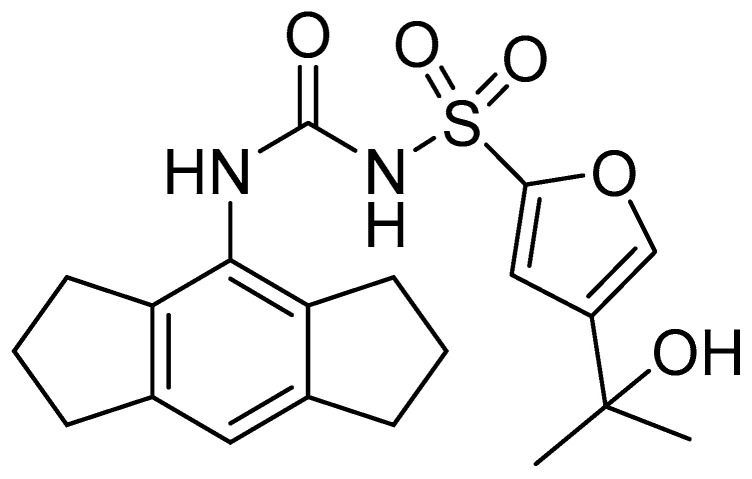
Structure of **MCC950**.

To assess the therapeutic potential of the NLRP3 inhibitor **MCC950** (Figure 6) on HL produced by systemic inflammation, Ma et al. constructed a mouse model with conditional expression of the CAPS-associated NLRP3 D301N gain-of-function mutation specifically in cochlea-resident CX3CR1 macrophages [6]. The susceptibility of these CAPS mice to inflammation-induced HL was subsequently evaluated under both local and systemic inflammation conditions [6]. Following LPS treatment in the middle ear cavity, NLRP3 mutant mice showed severe cochlear inflammation, inflammasome stimulation, and HL. However, this middle ear injection model produced substantial HL in control animals, resulting in inflammation-independent HL, likely attributable to procedural injury to the ear tissues. Next, a systemic LPS injection model was optimized and elicited pronounced HL in NLRP3 mutant mice but not in control animals. Repeated administration of low-dose LPS triggered peripheral inflammation, leading to disruption of the blood–labyrinth barrier, the infiltration of macrophages into the cochlea, and activation of the cochlear inflammasome in an NLRP3-dependent manner. Note also that both infiltrating macrophages and resident cochlear macrophages contributed to the development of peripheral inflammation-induced HL in CAPS mice. In addition, NLRP3i **MCC950** strongly reduced systemic LPS-induced HL and inflammatory manifestations in NLRP3 mutant animals. Collectively, these findings demonstrate that CAPS-mediated NLRP3 mutations are critical drivers of peripheral inflammation-mediated HL in this mouse model and support the therapeutic potential of selective NLRP3 inhibition for the treatment of inflammation-mediated SNHL [6].

## 5. Conclusions

Here, we have summarized the reported literature on NLRP3 in HL and discussed how NLRP3 influences and affects the disease development process. The opportunity for medication-based therapy for HL paves the way to its treatment. To summarize, the results presented above suggest that new drugs targeting mitochondrial dysfunction and modulating pathways via NLRP3 inhibition hold great therapeutic promise for the treatment of HL and related HL diseases.

## Data Availability

No new data was generated in this study.

## Abbreviations

The following abbreviations are used in this manuscript:

ABR	Auditory brainstem response
AID	Autoinflammatory disorders
ARHL	Age-related hearing loss
ASC	Apoptosis-associated Speck-like protein
C57BL/6J	C57 black 6; inbred strain of laboratory mouse
CAPS	Cryopyrin-associated periodic syndromes
CI	Cochlear implantation
CIAS1	Cold-induced autoinflammatory syndrome 1
CINCA	Chronic infantile neurological, cutaneous, and articular syndrome
CMV	Cytomegalovirus
CNS	Central nervous system
DFNA34	Deafness, autosomal dominant 34, with or without inflammation
ELISA	Enzyme-linked immunosorbent assay
ERK	Extracellular signal-regulated kinase
FCAS	Familial cold auto-inflammatory syndrome
FLAIR	Fluid-attenuated inversion recovery
GSDMD	Gasdermin D
HEI-OC-1	House ear institute-organ of Corti 1
HL	Hearing loss
IL-1β	Interleukin-1β
IgG1	Immunoglobulin G1
IL-1is	IL-1 inhibitors
IL-1Ra	IL-1 receptor antagonist
IL1R2	IL-1 receptor type 2
JNK	c-Jun N-terminal kinase
KM	Kanamycin
LRR	Leucine-rich repeat
LPS	Lipopolysaccharide
NGS	Next-generation sequencing
NEK7	never in mitosis A (NIMA)-related kinase 7
NIHL	Noise-induced hearing loss
NLRP3	NLR family pyrin domain containing 3
NLRP3i	NLRP3 inhibitors
NOMID	Neonatal-onset multisystem inflammatory disease
MC	Marginal cell
MCMV	Murine CMV
MD	Meniere’s disease
MEFV	Mediterranean fever
MLK	Mixed lineage kinase
MRI	Magnetic resonance imaging
MWS	Muckle–Wells syndrome
PCR	Polymerase chain reaction
Pou4f3	POU domain, class 4, transcription factor 3
PYD	Pyrin domain
qRT-PCR	Quantitative reverse transcription polymerase chain reaction
ROS	Reactive oxygen species
RT-PCR	Reverse transcription-polymerase chain reaction
SAA	Serum amyloid A
SGK1	Serum/glucocorticoid-inducible kinase 1
SGN	Spiral ganglion neurons
SNHL	Sensorineural hearing loss
TNF	Tumor necrosis factor
TUNEL	Terminal deoxynucleotidyl transferase dUTP nick-end labeling
TXNIP	Thioredoxin-interacting protein
UCB	Unconjugated bilirubin
VS	Vestibular schwannoma
WB	Western blot
